# Neuritogenic glycosaminoglycan hydrogels promote functional recovery after severe traumatic brain injury

**DOI:** 10.1088/1741-2552/ad5108

**Published:** 2024-06-27

**Authors:** Nathan Gonsalves, Min Kyoung Sun, Pradeep Chopra, Charles-Francois Latchoumane, Simar Bajwa, Ruiping Tang, Bianca Patel, Geert-Jan Boons, Lohitash Karumbaiah

**Affiliations:** 1 Regenerative Bioscience Center, University of Georgia, Athens, GA, United States of America; 2 Division of Neuroscience, Biomedical and Translational Sciences Institute, University of Georgia, Athens, GA, United States of America; 3 Complex Carbohydrate Research Center, University of Georgia, Athens, GA, United States of America; 4 Edgar L. Rhodes Center for Animal and Dairy Science, College of Agriculture and Environmental Sciences, University of Georgia, Athens, GA, United States of America; 5 Department of Chemistry, University of Georgia, Athens, GA, United States of America; 6 Department of Chemical Biology and Drug Discovery, Utrecht Institute for Pharmaceutical Sciences, and Bijvoet Center for Biomolecular Research, Utrecht University, Utrecht, The Netherlands

**Keywords:** SPAAC click chemistry, chondroitin sulfate glycosaminoglycans, chimeric peptide, reach-to-grasp function, traumatic brain injury

## Abstract

*Objective.* Severe traumatic brain injury (sTBI) induced neuronal loss and brain atrophy contribute significantly to long-term disabilities. Brain extracellular matrix (ECM) associated chondroitin sulfate (CS) glycosaminoglycans promote neural stem cell (NSC) maintenance, and CS hydrogel implants have demonstrated the ability to enhance neuroprotection, in preclinical sTBI studies. However, the ability of neuritogenic chimeric peptide (CP) functionalized CS hydrogels in promoting functional recovery, after controlled cortical impact (CCI) and suction ablation (SA) induced sTBI, has not been previously demonstrated. We hypothesized that neuritogenic (CS)CP hydrogels will promote neuritogenesis of human NSCs, and accelerate brain tissue repair and functional recovery in sTBI rats. *Approach.* We synthesized chondroitin 4-*O* sulfate (CS-A)CP, and 4,6-*O*-sulfate (CS-E)CP hydrogels, using strain promoted azide-alkyne cycloaddition (SPAAC), to promote cell adhesion and neuritogenesis of human NSCs, *in vitro*; and assessed the ability of (CS-A)CP hydrogels in promoting tissue and functional repair, in a novel CCI-SA sTBI model, *in vivo. Main results.* Results indicated that (CS-E)CP hydrogels significantly enhanced human NSC aggregation and migration via focal adhesion kinase complexes, when compared to NSCs in (CS-A)CP hydrogels, *in vitro*. In contrast, NSCs encapsulated in (CS-A)CP hydrogels differentiated into neurons bearing longer neurites and showed greater spontaneous activity, when compared to those in (CS-E)CP hydrogels. The intracavitary implantation of (CS-A)CP hydrogels, acutely after CCI-SA-sTBI, prevented neuronal and axonal loss, as determined by immunohistochemical analyses. (CS-A)CP hydrogel implanted animals also demonstrated the significantly accelerated recovery of ‘reach-to-grasp’ function when compared to sTBI controls, over a period of 5-weeks. *Significance.* These findings demonstrate the neuritogenic and neuroprotective attributes of (CS)CP ‘click’ hydrogels, and open new avenues for the development of multifunctional glycomaterials that are functionalized with biorthogonal handles for sTBI repair.

## 1. Introduction

Severe traumatic brain injuries (sTBI) result from blunt force or penetrating trauma to the brain. In the United States alone, about 69,473 TBI-related deaths occurred in 2021 [1]. Nearly 50% of sTBI survivors experience severe functional deficits and a high risk of death within 5-years of sustaining the injury [2, 3]. Despite early neurocritical care and surgical interventions, there are no efforts to remedy the long-term tissue volume and functional losses experienced by sTBI patients [4, 5].

The brain extracellular matrix (ECM) provides structural and functional support to neuronal networks, and constitutes approximately one-fifth of the total brain volume [6–9]. A distinctive feature of the brain ECM is the compartmentalization of fibrous proteins and proteoglycans in different brain regions. Heparan sulfate proteoglycans (HSPGs), alongside fibrous matrix proteins such as collagen, fibronectin, and laminin, are localized to the basement membrane, with chondroitin sulfate proteoglycans (CSPGs), hyaluronan, and a few fibrous proteins being distributed more abundantly in the neural interstitial matrix [8, 10]. Although fibrous ECM proteins are relatively less abundant in the brain ECM when compared to proteoglycans, they play important roles in providing structural support, mediating cell–cell interactions, and integrin-mediated adhesion of neural cells [11, 12]. CS glycosaminoglycans are laterally emanating side chains attached to Serine/Threonine residues on CSPG core proteins. They possess multivalent attributes, and can interact with multiple growth factors to potentiate cell signaling, neuroplasticity, axonal guidance, and neuronal growth and repair after brain injury [9, 13–19].

Tissue-engineered CS hydrogels have been demonstrated to promote neuroprotection, potentiate endogenous NSC renewal and mediate cellular and functional repair after sTBI [14, 20, 21]. Previous reports using rodent TBI models have suggested that the period between 4–5 weeks post-TBI constitutes a ‘sub-acute’ period after sTBI induction when animals first demonstrate sustained functional deficits [21–23]. Evidence also suggests that this timepoint is useful to determine treatment effects and differences between treated and untreated animal cohorts. Despite previous evidence, the functional characterization of CS hydrogels carrying neuritogenic chimeric peptides (CP), and their ability to promote neuritogenesis and functional recovery after sTBI has not yet been demonstrated.

We hypothesized that CS hydrogels carrying neuritogenic integrin binding peptides will promote neuritogenesis and accelerate functional recovery in sTBI rats, sub-acutely. We used strain promoted azide-alkyne cycloaddition (SPAAC) click chemistry [24, 25] to create multifunctional CS-hydrogels. We incorporated a previously designed chimeric peptide (CP) containing laminin derived RGDS and IKVAV sequences, which have been shown to promote NSC growth, migration and differentiation as well as neuritogenesis through integrin binding [26–29]. We synthesized (CS-A)CP and (CS-E)CP constructs and characterized them using ^1^H^-^NMR and rheological testing of hydrogel mechanical properties. Hydrogel topography and microarchitecture were characterized by scanning electron microscopy (SEM). We quantified the adhesion, aggregation, migration, differentiation, and neuronal activity, *in vitro,* of hydrogel encapsulated human induced pluripotent neural stem cells (HIP-NSCs). We performed controlled cortical impact (CCI) induced sTBI and damage to the ‘reach-to-grasp’ circuit in the M2 or Rostral Forelimb Area (RFA) of the adult male Sprague Dawley rat brain, followed by acute suction ablation (SA) to remove contused tissue. [21, 30–32]. The effect of intracavitary (CS-A)CP hydrogel implantation was evaluated by weekly forelimb ‘reach-to-grasp’ function assessments, and terminal immunohistochemical analyses at the study end-point of 5-weeks after sTBI.

## 2. Materials and methods

### 2.1. In vitro

#### 2.1.1. Chemical synthesis

Detailed synthetic procedure, NMR, and MS characterization of Azide-PEG-amine linker (**S2**), DIBO-PEG-amine linker (**S3**) from Amine-PEG-Amine (**S1**) and regioselective sulfonation of CS-A (**1**) to obtain CS-E (**2**) [33] is provided in supplementary information.

##### 2.1.1.1. Preparation of azide-functionalized CS-A (**3**) and CS-E (**4**)

0.5 g of CS-A (**1**) was dissolved in a mixture of water/dimethyl sulfoxide (30 ml, 2/1, v/v). Next, 1-Ethyl-3-(3-dimethylaminopropyl) carbodiimide (0.2 g, 1.08 mmol, EDC, Thermo, MA) and N-hydroxysuccinimide (0.12 g, 1.08 mmol, NHS, Thermo, MA) were added and left stirring for an hour. A solution of (**S2**) (molar ratio to CS = 0.6, 0.1 g, 0.63 mmol) in DMSO (10 ml) was added dropwise, and the resulting reaction mixture was left stirring at room temperature. After 24-hours, the reaction mixture was dialyzed for 3-days against water using 1 kD MWCO dialysis tubing (Spectrum Laboratories Inc., CA). Dialyzed Azido-CS-A, thus obtained, was filtered using a 0.2 *µ*m filter (Thermo, MA) and lyophilized to afford the desired compound (**3**) as a white fluffy solid. Azido-CS-E (**4**) was synthesized from (**2**) using the same method in 2.1.1.1 described above.

##### 2.1.1.2. Preparation of DIBO-functionalized CS-A (**5**)

To the stirring solution of (**1**) (0.5 g) in mixture of water/DMSO (1/2, 30 ml), EDC (0.2 g, 1.08 mmol) and NHS (0.12 g, 1.08 mmol) were added and stirred for 1-hour at room temperature. To the above solution, (**S3**) (molar ratio to CS = 0.6, 0.26 g, 0.65 mmol) in DMSO (10 ml) was added dropwise and left stirring for another 24-hours. Next, the reaction mixture was dialyzed for 3-days against water using 1 kD MWCO dialysis tubing (Spectrum Laboratories Inc., CA), filtered (0.2 *µ*m) and lyophilized to afford the desired compound (**5**) as a white fluffy solid.

##### 2.1.1.3. Synthesis of chimeric peptide (CP)

Azide functionalized chimeric peptide (CP) containing integrin binding sequences, RGDS and IKVAV, were synthesized on a rink amide resin (Novabiochem, San Diego, CA, 0.1 mmol) by established protocols [24] on a CEM Liberty Automated Microwave Peptide Synthesizer using standard Fmoc-protected amino acids. A terminal azide group was introduced by using Fmoc-Lys(N_3_)-OH amino acid. Final purification of CP was attained by reverse-phase high-pressure liquid chromatography (RP-HPLC) and characterized using mass spectrometry (MS-MALDI-ToF, Applied Biosystems 5800).

#### 2.1.2. Hydrogel preparation, characterization and functional testing

##### 2.1.2.1. Hydrogel preparation and characterization

Lyophilized DIBO-CS-A (**5**) and Azido-CS-A (**3**) or CS-E (**4**) were each dissolved in neurobasal medium (Thermo, MA) to achieve a 10% wt/v stock concentration. To obtain stable (CS-A)CP and (CS-E)CP hydrogels, we added 1 mM final concentration of CP (from a 10 mM stock) to 2.5% DIBO-CS-A (from 10% stock) (**5**), crosslinked them with 1% Azido-CS-A (from 10% stock) (**3**) or 1% Azido-CS-E (from 10% stock) (**4**), and mixed gently by pipetting to initiate gelation at room temperature. Stable hydrogels were formed within 10 min of incubation, as confirmed by using the vial tilting method [25]. Accordingly, all hydrogel components were added to a snap cap tube or glass vial, and the vial containing hydrogel was tilted at Time = 0 min to assess liquidity. The process was repeated at Time = 10 min to demonstrate gelation and stable hydrogel formation. Characterization of CS-hydrogels were performed through Scanning Electron Microscopy (SEM), Rheology and Swelling and Degradation studies (Supplementary figure 1(B)).

##### 2.1.2.2. Hydrogel encapsulation of hNSCs

(CS-A)CP and (CS-E)CP hydrogels were formed as described above. For NSC live-cell tracking and migration, 1 × 10^5^ hNSCs (ThermoFisher) were encapsulated in (CS-A)CP and (CS-E)CP hydrogels, respectively. For vinculin/FAK staining, and aggregation potential, 3 × 10^5^ hNSCs were encapsulated in each hydrogel type. 5 × 10^6^ cells ml^−1^ hNSCs were seeded in dishes in order to yield a good number of differentiated neurons for calcium imaging experiments. For individual hydrogel encapsulated experiments, appropriate cell numbers, as described above, were mixed with the hydrogel precursors, and the mixture was quickly dispensed onto a glass bottom dish for gelation. Cell encapsulated hydrogels were overlaid with expansion media and incubated at 37 °C/5% CO_2_ for the respective assays.

##### 2.1.2.3. Immunocytochemistry

hNSCs encapsulated in (CS-A)CP and (CS-E)CP hydrogels were fixed in 4% paraformaldehyde (PFA) with 0.4 M sucrose in PBS, and blocked using blocking buffer (4% goat serum, 0.5% Triton-X 100 in PBS). hNSCs were stained with primary antibodies: Sox1 (R&D Systems, MN), Nestin (Millipore, CA), Focal Adhesion Kinase (FAK, Novax, CA), Vinculin (Sigma, MO), and Class III *β*-tubulin (Millipore, CA) overnight at 4 °C. Next day, hydrogels were blocked again in blocking buffer and stained for 3-hours with secondary antibodies (Alexa Fluor 488, 555 and 647; Thermo, MA). Hydrogels were finally incubated with nuclear stain (DAPI; Thermo, MA) in PBS for 10 min, and mounted with Fluoromount-G (Southern Biotech, AL). Cells were imaged using Zeiss LSM 710 confocal microscope (Zeiss, Germany). Fiji [34] was used to process raw images obtained for co-localization (Coloc2 plugin) of Vinculin/FAK and Sox1/DAPI. Neurite length was calculated using the published automated quantification method, Neurite Tracer [35]. Aggregation size based on Nestin^+^ area and circularity based on Class III *β*-tubulin were calculated using built-in functions in Fiji.

##### 2.1.2.4. Cell migration assay

hNSCs in (CS-A)CP and (CS-E)CP hydrogels were stained with Hoechst (1 µg ml^−1^; Thermo, MA) and DiO (5 µg ml^−1^; Thermo, MA) for 20 min in serum-free neurobasal media and rinsed twice in expansion media before seeding on a 35-mm single glass-bottom dish. Immediately upon gelation (10 min), hNSCs were overlaid with expansion media and imaged over 1-hour using an Inverted Fluor Polarizing Microscope (Leica DM IRBE; Leica Microsystems, Buffalo Grove, IL). Images were obtained every 10 min. Cells were maintained in a Tokai live cell culture microscope stage top incubator at 37 °C with a flow of 5% CO_2_ medical grade air mixture (Airgas, PA) during the recording session. Migration speed was calculated using Volocity software (PerkinElmer, MA).

##### 2.1.2.5. Calcium imaging

hNSCs were differentiated in media without FGF2 for 7-days before being overlaid on top of crosslinked (CS-A)CP and (CS-E)CP hydrogels in 2D on 35-mm single glass-bottom dish. After 48-hours, a stock solution of 50 *µ*g of Fluo 4-AM (Thermo, MA) dissolved in 20% F127 pluronic acid in DMSO (v/v) was prepared. Spent media was replaced with pre-warmed loading solution and incubated for 20 mins. Cells were then transferred to Brainphys^®^ only solution for recording. Calcium recording was performed using Leica DM-IRBE inverted microscope with a TRITC filter (Ex/Em: 561/576 nm) while maintaining cells in a Tokai live cell culture microscope stage top incubator as previously described. Images were obtained for 5 mins at 1 frame per sec (fps) with 0.5 s exposure. Recorded time lapses were processed using MATLAB^®^ 2021. Cell soma were detected using maximum intensity projection, and regions of interest (ROI) were automatically defined using custom-made scripts in MATLAB^®^ using the image analysis toolbox. Calcium activity traces were extracted from ROI and the spikes were detected using normalized Δ*F*/*F* and peak detection using MATLAB^®^.

### 2.2. In vivo

#### 2.2.1. Animals and ethics statement

Sprague-Dawley rats were obtained from Charles River Laboratories (*n* = 23, 4–6 weeks old, males only; and randomly assigned to each treatment: control craniotomy with no injury (Sham group: *n* = 6), CCI-SA (sTBI group: *n* = 9), and CCI-SA implanted with (CS-A)CP hydrogel [CCI-SA-(CS-A)CP group: *n* = 8]. All rats were individually housed with ad libitum access to food and water and following a reverse 12 h-light cycle (light OFF 7:00-19:00, Light ON 19:00-7:00) in a room maintained at 70% humidity and 23 °C. All procedures on animals were approved by the Institutional Animal Care and Use Committee (IACUC) of the University of Georgia, and protocols were performed in accordance with the Guide for the Care and Use of Laboratory Animals published by the National Institution of Health (NIH).

#### 2.2.2. Surgical procedures

##### 2.2.2.1. Controlled cortical impact (CCI) and suction ablation (SA) surgeries

Before surgery, each animal was anesthetized with 5% isoflurane gas and buprenorphine was injected subcutaneously (0.05 mg kg^−1^; Henry Schein) followed by 2% Lidocaine HCL injected locally at the injection site. At Day 0, animals were placed on a stereotaxic frame attached to a temperature-controlled heating pad (37 °C) with their scalp shaved and sanitized (Chlorhexidine and 70% EtOH). A 5-mm (diameter) craniotomy was performed on top of the RFA or M2 motor cortex (AP: +3.0 mm; ML: +2.5 mm, relative to bregma) using a 1-mm-drill bit fitted to an electronic drill. A CCI injury to damage the reach-to-grasp circuit was subsequently delivered to the epicenter of the craniotomy. Absorbable gelatin (Gel foam, Ethicon) was applied to the injury site, and sterile cotton swabs were used to remove excess blood. Skin flaps were sutured together, closing the wound and triple antibiotic cream was applied on the sutured skin. Animals were allowed to recover and transferred to the housing room.

After 48-hours, each animal was re-anesthetized with 5% isoflurane gas and buprenorphine was re-injected subcutaneously (0.05 mg kg^−1^; Henry Schein). A sterile custom 3D-tissue biopsy punch (3-mm diameter) attached to a vacuum line was used to resect contused tissue at 2-mm depth. The lesion area was sanitized with sterile saline and gel foam. Each animal in the hydrogel treatment cohort received a 20 *µ*l intracavitary (CS-A)CP hydrogel implant immediately after SA. Skin flaps were sutured together to close the wound, and triple antibiotic cream was applied on the sutured skin. Animals were allowed to recover and transferred to the housing room. Detailed descriptions of CCI and SA injury model and parameters are provided in supplementary methods section.

##### 2.2.2.2. Preparation and intralesional delivery of (CS-A)CP hydrogel

As described for *in vitro* hydrogel preparation, (CS-A)CP hydrogel precursors were mixed with sterile Hank’s Balanced Salt Solution (HBSS) (Corning, NY) and allowed to gel for 10 min. Following suction ablation at 48-hours, hydrogel was loaded in a 50 *μ*l Hamilton syringe (fitted with a blunt 21-gauge tight lock stainless steel needle) and 20 *μ*l was delivered intralesionally (depth: 2-mm; speed: 2 *μ*l min^−1^ for 10 min).

#### 2.2.3. Brain tissue preparation and immunohistochemistry

Animals were sedated using Ketamine (65 mg kg^−1^ and Xylazine (7.5 mg kg^−1^) and transcardially perfused with Phosphate Buffered Saline (PBS, pH 7.2) for 20 min. The extracted whole brain was placed on a coronal, 1-mm, brain matrix (Ted Pell Inc.) and the rostral half of tissue (+4.5 mm to +0.5 mm, relative to bregma) was sliced and flash frozen in liquid nitrogen, and stored in −80° until cryosectioned. A total of 200 coronal sections (14 *µ*M thick) were serially collected from rostral side, moving caudally on 50 slides (4 sections per slide). 3 slides per animal (140 *µ*M apart) were fixed using 4% paraformaldehyde in 0.4 M sucrose, blocked using 5% BSA in PBST (0.5% Triton-X 100 in PBS) and incubated with primary antibody, (see supplementary table 1) overnight, at 4 °C. Next day, slides were blocked again in blocking buffer, followed by secondary antibody staining for 3-hours, at room temperature. Finally, slides were stained with 1 mg ml^−1^ Hoechst (Invitrogen 33342, Trihydrochloride, Trihydrate, Dilution 1:200), airdried and cover slipped with Fluoromount-G Mounting Medium (Southern Biotech, AL) and stored at −20°C until imaged. 10X tiled images and 40X oil images were taken using Zeiss LSM 880 confocal microscope (Zeiss, Germany). It must be noted that excised brain tissue from one animal belonging to the CCI-SA group was ruptured while flash freezing and IHC staining was not performed on that animal.

##### 2.2.3.1. Skilled reach task assay

Chronic recovery of forelimb-specific function was assessed using the skilled reach task (SRT) [36]. Animals were placed in a transparent plexiglass box (25 × 40 × 40 cm) with side openings (forced left-side training: slit width: 0.8 cm, slot distance from edge: 1 cm). Rats were subjected to mild food deprivation prior to behavioral testing (3-hours, body weight drop <1%). During a training or a test session, rats were allowed to attempt retrieving a maximum of 20 food pellets, (Bioserv, 20 mg) for a maximum duration of 20 min. Rats were trained for 2-weeks of forced left (FL) training with food pellets placed on the left side of the box (side opening), allowing retrieval using only the left paw. A rat was deemed successfully trained if it was able to retrieve 20 food pellets under 20 min for two consecutive sessions. Rats were further tested once a week at weeks 1, 2, 3, 4 and 5, post-TBI. It must be noted that one animal from the CCI-SA-(CS-A)CP group did not complete 5 weeks of SRT due to loss of motivation and was removed from the behavioral analysis.

### 2.3. Statistics

GraphPad Prism software 10.0.2 was used to plot graphs and to evaluate the significance between groups either using the Student t-test (normal data distribution) or the Mann**–**Whitney Rank Sum-test (when failed normal distribution) for *in vitro* data. For *in vivo* immunohistochemical analysis, Kruskal–Wallis Test with uncorrected Dunn’s post hoc comparison was used. Two-Way repeated measures ANOVA with Tukey’s post coc comparison was used for behavioral data. All numeric values were presented as Mean ± SEM. All *in vitro* assays were performed in 3 biological replicates, except aggregation area and size determinations, which were performed in duplicate. For all tests, *p < 0.05 was considered significant.

## 3. Results

### 3.1. Chemical synthesis of CS-hydrogels

**Figure 1.**
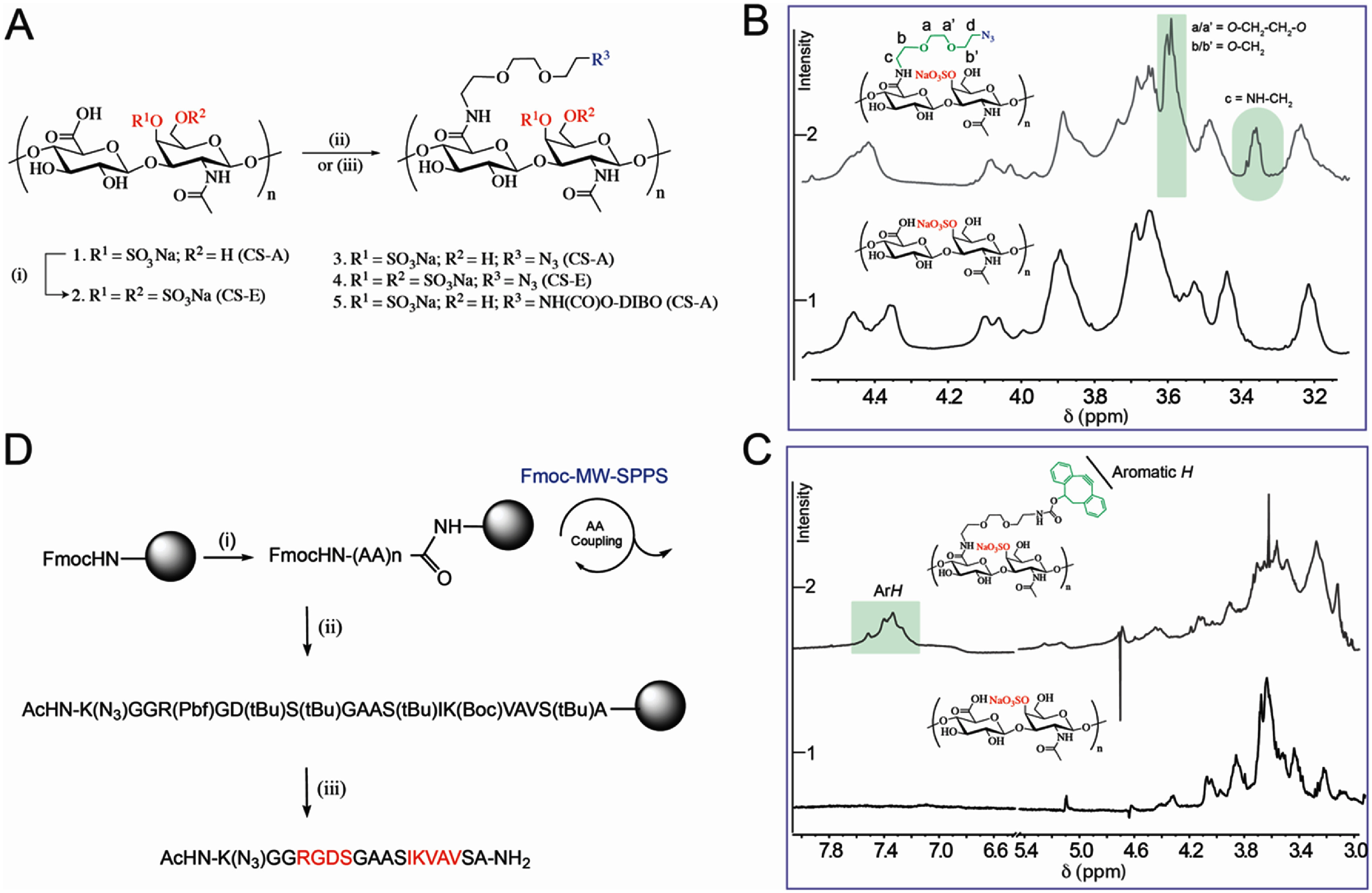
**Synthesis of CS-hydrogels.** (A) Synthesis of azide and DIBO functionalized CS-A and CS-E. Reagents and conditions: (i) SO_3_. Pyridine complex, 60 °C, 16 h; (ii) S2, EDC, NHS, DMSO/H_2_O (4/1), RT, 24 h; (iii) S3, EDC, NHS, DMSO/H_2_O (4/1), RT, 24 h; (B) ^1^H-NMR overlay of azide functionalized and unmodified CS-A, highlighted region represents characteristic protons of azido-amine linker. The degree of azide functionalization on CS-A backbone was estimated to be about 35%–45% based on ^1^H-NMR integrations; (C) ^1^H-NMR overlay of dibenzo cyclooctyne (DIBO) functionalized and unmodified CS, highlighted region represents characteristic aromatic protons of DIBO, the degree of azide functionalization was estimated to be about 10%–15% based on ^1^H-NMR integrations; (D) Microwave-assisted solid phase synthesis (MW-SPPS) of Chimeric peptide (CP). Reagents and conditions: (i) 20% 4-methylpiperidine, DMF, MW, 3 min; (ii) Fmoc-AA-OH, HOBt, HBTU, DIPEA, DMF, MW, 5 min; and (iii) TFA/H_2_O/TIPS (95/2.5/2.5), RT, 2 h.

We used SPAAC chemistry to prepare click crosslinked CS hydrogels (figure 1(A)). We synthesized azide functionalized CS-A (**3**) and CS-E (**4**), to crosslink with a DIBO functionalized CS-A (**5**). To avoid any steric issues, we chose a small ethylene glycol linker (**S1**) to modify the CS backbone. By stochiometric control, one amine group of ethylene glycol linker (**S1**) was modified with either azide (**S2**) [37 **]** or DIBO (**S3**) leaving the other amine group free for conjugation to CS backbone (Supplementary figure 1(A)). The free amine group of the linker (**S2** or **S3**) was coupled to the carboxylic acid group of GlcA of CS-A (**1**) or CS-E (**2**) using carbodiimide chemistry to afford **3** (from **1**), **4** (from **2**) and **5** (from **1**) in high yields (figure 1(A)). The efficiency of coupling and degree of functionalization was determined using 1D/2D nuclear magnetic resonance (NMR) experiments. A stack plot of ^1^H-NMR spectra of **3** and **1** (figure 1(B)) and **5** and **1** (figure 1(C)) highlights characteristic peaks. An acceptable level of ∼35%–45% functionalization was observed, for **3** (or **4**) and ∼10%–15% for **5**. The integral ratio was calculated using the following formula: (A) % Functionalization of CS-DIBO = [(Integration value of aromatic protons at 7.5 ppm/8)/(integration value of acetyl proton/3)]*100. (B) % Functionalization of CS-Azide = [(Integration value of PEG-CH_2_ protons at 4.5 ppm/2)/(integration value of acetyl proton/3)]*100. Additionally, an azide modified chimeric peptide (CP) [(AcHN-K(N_3_)GGRGDSGAASIKVAVSA-NH_2_)] containing laminin-derived integrin binding peptide motifs (RGDS and IKVAV) was synthesized (figure 1(D)) and incorporated at 1 mM final concentration to the hydrogel to promote integrin recognition and cell adhesion.

### 3.2. 3.5% CS-A hydrogels mimic the biomechanical properties of native brain tissue

In order to test crosslinking efficacy of CS-A hydrogel components (**3**, **4** and **5**), 2% or 2.5% of **3** (or **4**) was mixed with 1% of **5** in a glass vial and incubated at room temperature separately. A stable hydrogel was formed within 10 min, as judged by the vial-tilting method.

To confirm biocompatibility of the crosslinked hydrogel, mechanical properties were examined prior to cell encapsulation. Lyophilized hydrogels were imaged using scanning electron microscopy (SEM). 3.5% CS-A hydrogel displayed a denser structural network with 25.23 ± 17.51 *µ*m (Supplementary figure 1(b-A)) as determined by the average pore sizes (diameter), while 3% CS-A hydrogel had an average pore size of 17.18 ± 12.01 *µ*m (Supplementary figure 1(b-B)). Next, CS-A hydrogels were tested for their rheological properties (Supplementary figure 2(C)). The storage modulus of the 3.5% CS-A hydrogel ranged between 600 and 1100 Pa as the frequency sweep increased, which closely mimics normal healthy brain tissue stiffness [38, 39]. However, the storage modulus of the 3% CS-A hydrogel remained relatively low, barely reaching 400 Pa at 100 rad s^−1^. Over a period of 14-days, *in vitro,* the 3.5% CS-A hydrogels did not swell excessively, as determined by calculating the swelling ratio (87.91 ± 3.43%). The gels also degraded in the presence of hyaluronidase treatment. (Supplementary figure 2(D)). Based on these findings, the 3.5% CS-A hydrogels were selected for further functional testing.

### 3.3. CS-hydrogels facilitate hNSC migration

**Figure 2.**
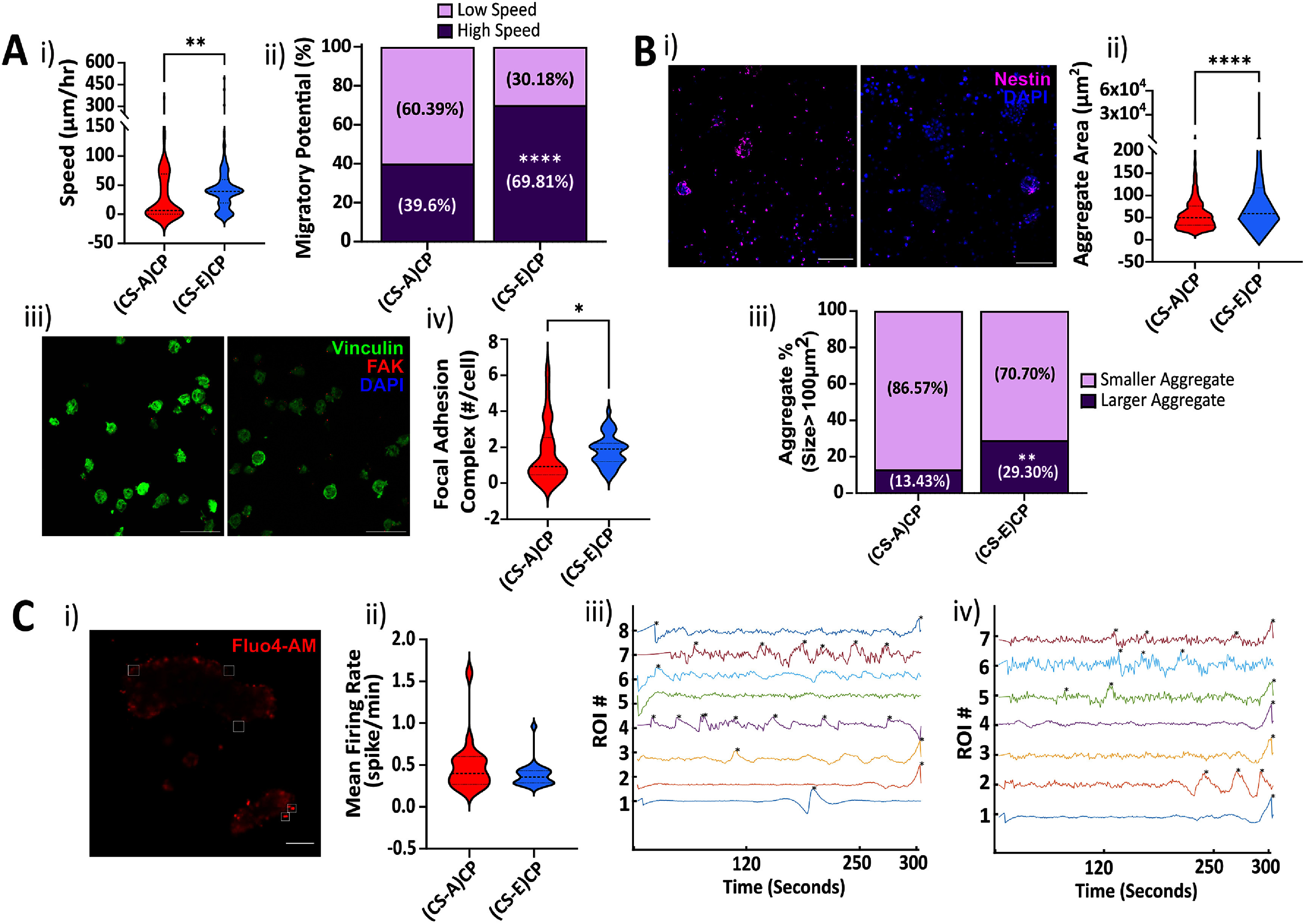
**Chimeric Peptide functionalized CS-hydrogels regulate undifferentiated NSC function and activity of differentiated neurons, *in vitro*.** (A) Migratory Potential—(i) Undifferentiated NSC migratory speed (*µ*M h^−1^) in (CS-A)CP and (CS-E)CP hydrogels were measured over a period of 1 h. Mann–Whitney Rank Sum Test. **p = 0.0049 (ii) NSCs with high migratory potential (migratory speed > 30 *μ*m h^−1^) over entire NSC population within the ROI was calculated. Fisher’s Exact Test. ****p< 0.0001 (iii) Representative images of NSCs in (CS-A)CP (left) and (CS-E)CP (right) hydrogels stained with Vinculin (green), FAK (red) and DAPI (blue). Scale bar: 30 *µ*m. (iv) The number of focal adhesion complexes/cell were calculated based on the number of FAK puncta colocalized with vinculin. Mann–Whitney Rank Sum Test. *p < 0.05 (B) Aggregation Potential—Undifferentiated NSCs cultured in (i) (CS-A)CP (left) and (CS-E)CP (right) hydrogels stained with Nestin (magenta), and DAPI (blue). Scale Bar − 80 *µ*m. (ii) NSC aggregate area (*µ*m^2^) was calculated based on the confocal images of Nestin overlaid with DAPI. (iii) Aggregates larger than 100 *µ*m^2^ were binned as a population over entire NSC population. Fisher’s Exact Test. **p = 0.0087. (C) Activity of neurons differentiated from NSCs—(i) Snapshot of calcium signal detection in (CS-A)CP hydrogel. White squares show ROI around fluorescing cells. Scale Bar − 20 *µ*m. (ii) Mean firing rate of the active ROIs obtained from Fluo4-AM recordings in neurons from both hydrogels. Mann–Whitney Rank Sum Test. *p < 0.05. Representative extracted calcium traces of Fluo4-AM recording over 5 min in iii) (CS-A)CP and iv) (CS-A)CP hydrogels. *indicate the detection of a calcium spike from the Δ*F*/*F* processed traces.

In order to investigate migration potential of the hNSCs, migration speed of cells encapsulated in (CS-A)CP and (CS-E)CP rich hydrogels were calculated via live-cell imaging. 10 min post-encapsulation, Hoechst and DiO labelled hNSCs were imaged for 30 min to measure their migration trajectories, and the average speed was calculated. The average speed of hNSCs in CS-E hydrogel (54.55 ± 7.11 *µ*m h^−1^) was significantly increased (Mann–Whitney Rank Sum, **p = 0.0049) compared to the hNSCs in CS-A hydrogel (38.54 ± 5.80 *µ*m h^−1^) (figure 2(A-i)). Next, hNSCs were stained with Sox1, an early neuroectoderm marker. No statistically significant differences (Mann–Whitney Rank Sum Test, *p* = 0.1298) were observed between Sox1^+^ vs. DAPI expressing cell ratios in (CS-A)CP hydrogels compared to those in (CS-E)CP hydrogels. (Supplementary figure 2(A)). Viability of hNSCs encapsulated in 3.5% CS hydrogels was also largely unaffected (Supplementary figure 2(B); 81.6 ± 12 %).

hNSC migration speed greater than 30 *µ*m h^−1^ was categorized as a cell with high migratory potential. hNSCs in (CS-A)CP and (CS-E)CP hydrogels were binned into either high or low migratory potential cells (figure 2(A-ii)). (CS-E)CP hydrogels (69.81%) had almost twice as many cells with high migratory potential than (CS-A)CP hydrogels (39.6%; Fisher’s Exact Test: ****p < 0.0001). The number of focal adhesions (FAK/vinculin colocalization) per cell was examined upon crosslinking (figure 2(A-iii)). In accordance with the migration speed, hNSCs in (CS-E)CP rich hydrogels (median = 1.906) expressed a significantly higher number of focal adhesions than hNSCs in (CS-A)CP hydrogels on average (median = 0.933; Mann–Whitney Rank Sum, *p* = 0.033) (figure 2(A-iv)).

### 3.4. CS-hydrogels potentiate hNSC aggregation

At 5-days post-encapsulation, hNSCs were stained with the stem cell cytoskeletal marker, Nestin, to study hNSC distribution within the hydrogel (figure 2(B-i)). We observed that hNSCs formed significantly larger aggregates in (CS-E)CP rich hydrogels (median = 58.57; Mann–Whitney Rank Sum, ****p < 0.0001) (figure 2(B-ii)) with 29.30% of the aggregates having an area larger than 100 *µ*m^2^, when compared to 13.43% of aggregates larger than 100 *µ*m^2^ (Fisher’s Exact Test, **p = 0.0087) in (CS-A)CP rich hydrogels (median = 49.92) (figure 2(B-iii)).

### 3.5. CS-hydrogels support neuronal differentiation, neuritogenesis, and neuronal activity

We tested if the degree of sulfation in CS hydrogels affects the functionality of differentiated neurons (figure 2(C)). Neurons derived from hNSCs were seeded on crosslinked CS hydrogels and were allowed to form networks over a period of 48-hours. The average neuronal firing rate was calculated based on calcium spikes detected from Fluo4-AM imaging (figure 2(C-i)). Both (CS-A)CP and (CS-E)CP hydrogels supported neuronal activity with no statistically significant differences detected between both populations (Mann–Whitney Rank Sum, *p* = 0.2851) (figure 2(A-ii)–(iv)). However, cells in (CS-A)CP hydrogels demonstrated an overall higher mean firing rate.

Differentiated neurons were further stained with *β*-III-tubulin (B3T) and DAPI to quantify the neuritogenic potential of the CP (Supplementary figure 2(b–A)). No significant differences in neurite length was observed between neurons in (CS-A)CP (median = 2658 *µ*m) when compared to (CS-E)CP hydrogels (median = 1919 *µ*m, Mann–Whitney Rank Sum test, *p* = 0.1147) (figures 2(b–B) (i)). No significant differences were detected in the neurite stained area of neurons in two hydrogels (Mann–Whitney Rank Sum test, *p* = 0.9099) (figures 2(b–B) (ii)). Quantification of the circularity of differentiated neuron clusters in CS hydrogels indicated that neurites and cell bodies in (CS-E)CP rich hydrogels maintained a spherical shape and did not extend neurites beyond the cell clusters (median = 0.148). Neuritogenesis in the (CS-A)CP rich hydrogel was enhanced, and the circularity value of cell clusters was significantly reduced when compared to (CS-E)CP (median = 0.116; Mann–Whitney Rank Sum test, *p = 0.0499) (figures 2(b–B) (iii)).

### 3.6. Acute intracavitary implantation of (CS-A)CP hydrogel post-sTBI accelerates the recovery of forelimb motor function

**Figure 3.**
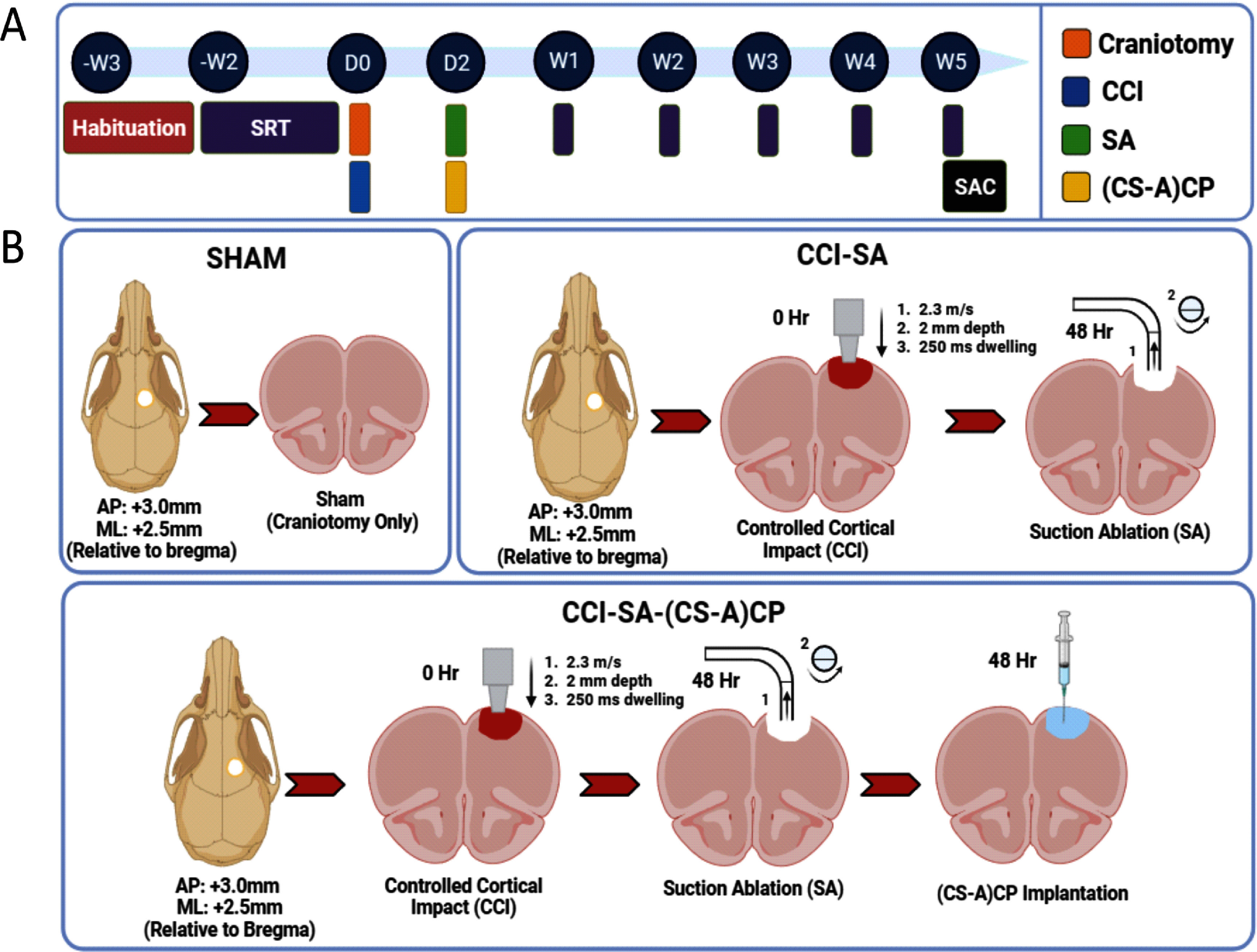
**Schematic of injury model and skilled reach task behavioral training**. (A) Experimental timeline for skilled reach task (SRT) behavioral training. Animals underwent 2-weeks of forced left (FL) pre-training followed by sTBI and (CS-A)CP implantation and post injury behavioral assessment for upto 5-weeks. (B) Schematic for injury paradigm across Sham, CCI-SA and CCI-SA-(CS-A)CP groups. 5-mm diameter craniotomy performed above the rostral forelimb area (RFA or M2 cortex; +2.5 mm ML, +3.0 mm AP, relative to bregma) of brain. Sham animals received craniotomy alone with no injury; CCI-SA animals received craniotomy and CCI at Day 0, and SA at Day 2; CCI-SA-(CS-A)CP animals received craniotomy and CCI at Day 0, and SA followed by intracavitary (CS-A)CP hydrogel implantation at Day 2. Figure created with Biorender.com.

**Figure 4.**
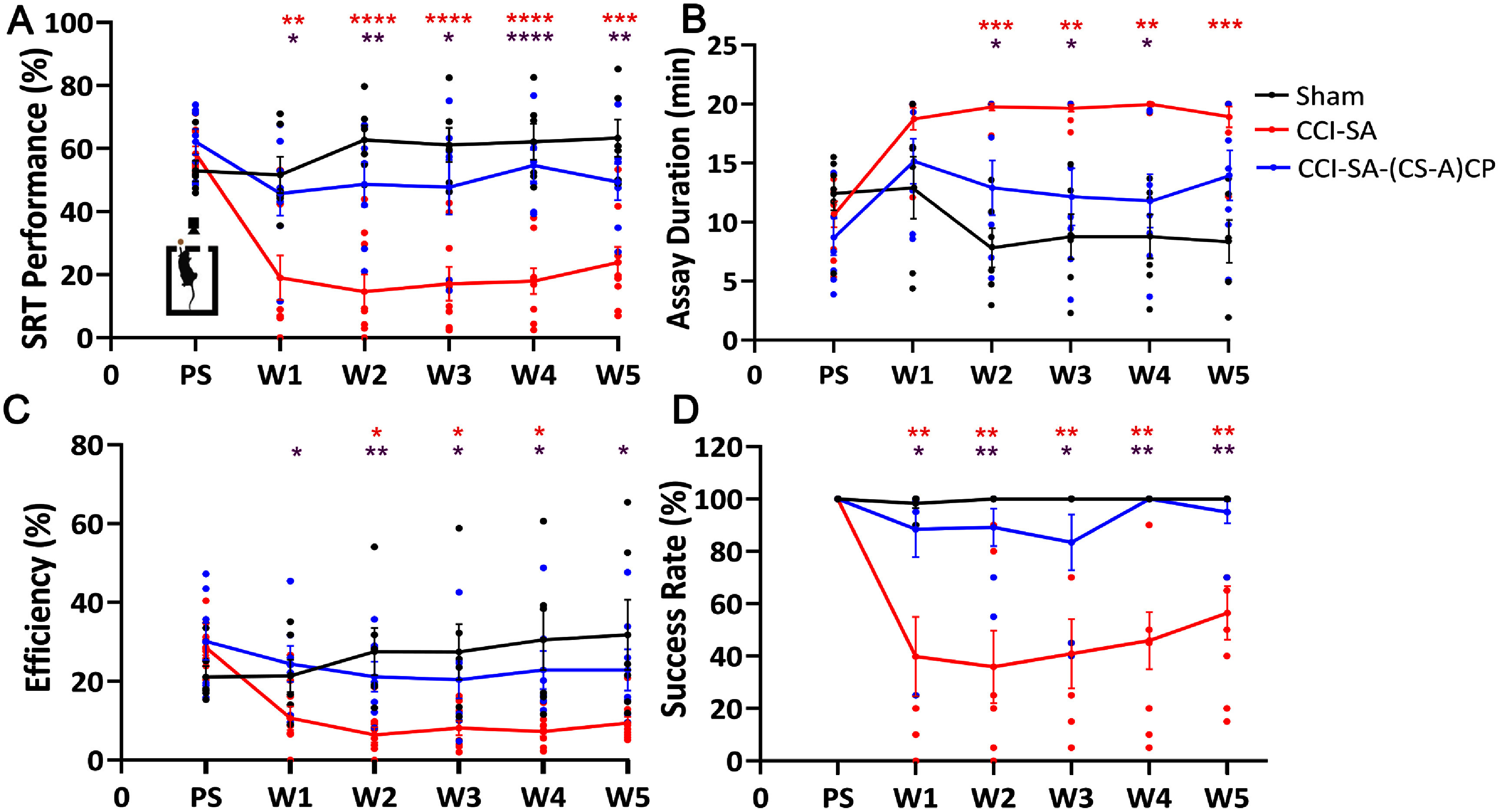
**(CS-A)CP hydrogel implants facilitate the recovery of forelimb motor function, over a period of 5-weeks after sTBI.** (A) Skilled reach task (SRT) performance was estimated as the average of percentage-normalized score obtained for assay duration, success rate and pellet retrieval efficiency. 100% indicates the best score possible to perform the assay. Sham (*n* = 6), CCI-SA (*n* = 9), and CCI-SA-(CS-A)CP (*n* = 7) groups after sTBI and treatment. (B) Assay duration is the time of completion of the task (e.g. 20 pellets retrieved successfully). Maximum duration: 20 min. (C) Efficiency is the number of pellets retrieved successfully using the left limb as a percentage of a total left limb attempts. (D) Success Rate (%) defined as total number of pellets retrieved/20 × 100. Repeated-measures ANOVA with Tukey’s Post Hoc Correction. *p < 0.05, **p < 0.01, ***p < 0.001 and ****p < 0.0001. Colored lines indicate group Mean ± SEM. Color-coded group comparisons indicate Sham versus CCI-SA (red), Sham versus CCI-SA-(CS-A)CP (blue), and CCI-SA versus CCI-SA-(CS-A)CP (purple).

We trained rats for 2-weeks (pre-injury) using forced left (FL) training on the skilled reach task (SRT) to assess ‘reach-to-grasp’ function for upto 5-weeks after sTBI. The injury was performed in the rostral forelimb area (RFA or M2 cortex, +3.0 mm AP, +2.5 mm ML, relative to bregma) to specifically damage left forelimb reach-to-grasp motor function (figure 3). Post-injury assessment of reach-to-grasp function was performed weekly for a period of 5-weeks. Our results demonstrate that CS-A(CP) hydrogel implanted rats showed SRT performance that was comparable to Sham controls, 1-week after sTBI. In contrast, a significant loss of forelimb motor function was observed in the CCI-SA animals at Week 1 (figure 4(A)). Significant differences in forelimb functional deficits persisted in CCI-SA animals through Week 5, with only transient improvement observed after Week 3. Hydrogel implanted animals showed significantly improved reach-to-grasp performance efficiency (figure 4(C)), reduced assay duration (figure 4(B)), and higher success rates (figure 4(D)) when compared to sTBI controls over a period of 5-weeks.

### 3.7. (CS-A)CP hydrogel implantation facilitates neuroprotection after sTBI

**Figure 5.**
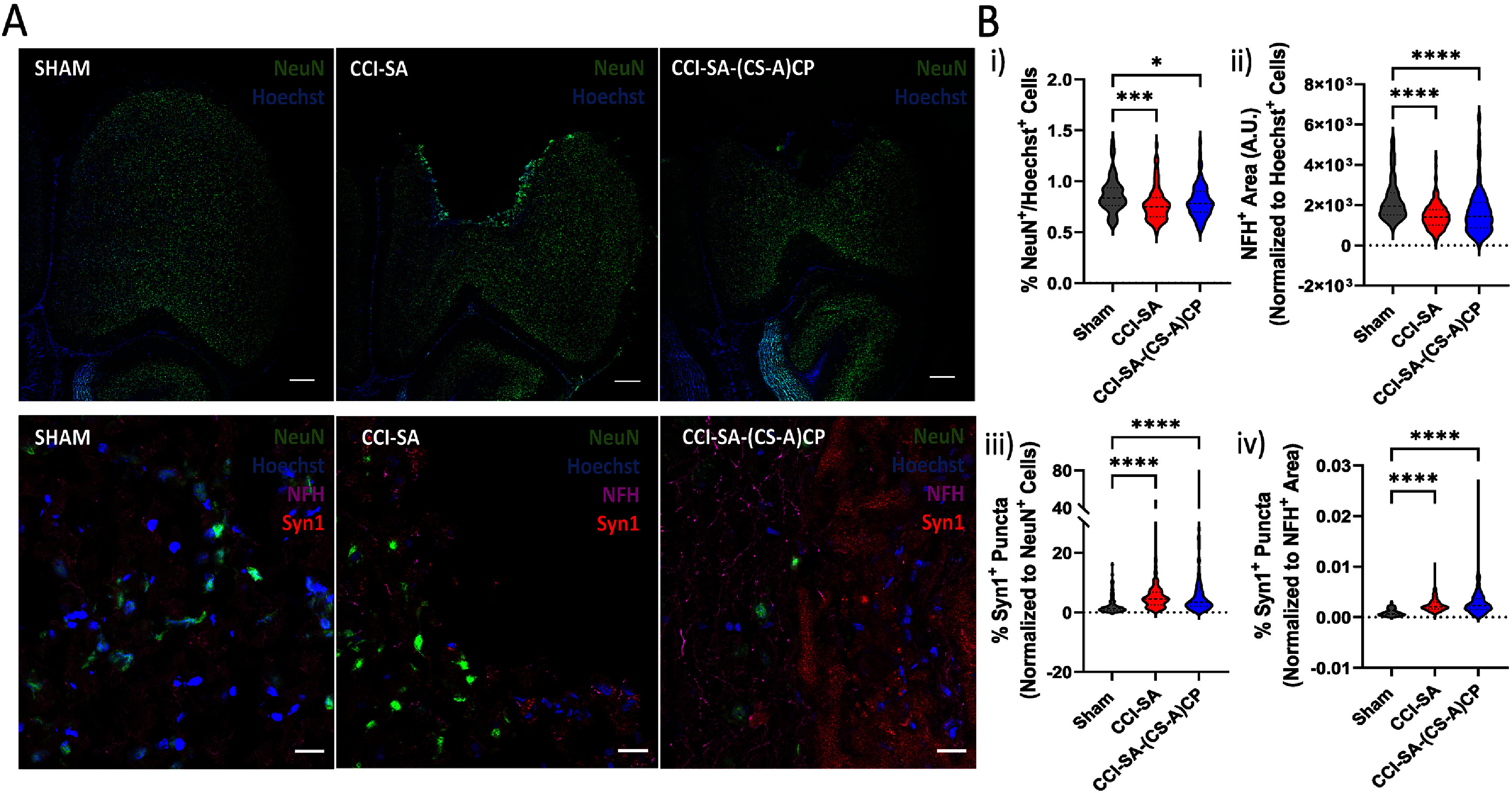
**(CS-A)CP hydrogel implant promotes neuroprotection, 5-weeks after sTBI.** (A) Representative 10X tiled confocal images from Sham (*n* = 6), CCI-SA (*n* = 8) and CCI-SA-(CS-A)CP (*n* = 8) animals stained for neuronal presence (NeuN^+^ in green) and nuclear marker (Hoechst^+^ in blue). Representative 40X oil images for respective groups stained for NeuN^+^ (green), Syn1^+^ (red), Neurofilament-heavy (NFH^+^) (magenta) and Hoechst^+^ (blue). 3 slides per animal containing 4 sections per slide were used for imaging and analysis. (B) Violin plot representing (i) % NeuN^+^/Hoechst^+^ cells from 10X tiled ipsilesional brain images, (ii) Perilesional NFH^+^ Area (A.U.) weighted on Hoechst^+^ cells from 40X oil images, (iii) Perilesional % Syn1^+^ puncta weighted on NeuN^+^ cells from 40X oil images, and (iv) Perilesional % Syn1^+^ puncta weighted on NFH stained area from 40X oil images. Kruskal–Wallis test with uncorrected Dunn’s test. *p < 0.05, **p < 0.01, ***p < 0.001, ****p < 0.001.

Terminal immunohistochemical analysis of brain tissue indicated that animals treated with (CS-A)CP hydrogels demonstrated enhanced neuritogenesis and neuroprotection after sTBI (figure 5(A)). Results demonstrated a higher neuronal presence as indicated by the greater percentage of NeuN^+^ neurons (NeuN^+^/Hoechst^+^ cells), in the hydrogel treated animals, compared to CCI-SA-sTBI animals (figure 5(B-i)). We also observed greater perilesional presence of axons and dendrites through neurofilament-heavy (NFH) staining (NFH^+^ area normalized to Hoechst^+^ cells) (figure 5(B-ii)). Hydrogel implanted animals also demonstrated higher percent perilesional synaptic vesicle (Syn1^+^ puncta normalized to NeuN^+^ cells and NFH^+^ area) presence, suggesting higher synaptic activity (figure 5(B-iii)–(iv)) compared to sTBI controls.

## 4. Discussion

CS glycosaminoglycan chains carry diverse sulfation patterns that possess the ability to bind and regulate growth factor signaling [13, 16, 40–42]. CS-A glycosaminoglycans are found in greater abundance in the adult mammalian brain and cortex [21, 43] when compared to CS-E (<3%) [44 **–** 46], which is more abundantly expressed in the brain germinal niche [41, 42]. The selective compartmentalization of CS sulfation patterns in the brain is reflective of their unique roles in promoting neuronal patterning and NSC maintenance, respectively. This also lends credence for multivalent CS ‘sulfation code’ dependent regulation of secreted factors and cellular responses [47]. Under homeostatic brain conditions, CSPGs carrying CS glycosaminoglycans are in close association with neuritogenic fibrous ECM proteins such as laminin and fibronectin. Both, CS-A and CS-E have been previously reported to inhibit neurite outgrowth and axonal guidance [13, 16, 40, 47–53]. However, the neuritogenic attributes of CS-A and CS-E hydrogels, functionalized with integrin binding peptides to mimic the function of fibrous proteins in the brain ECM, has not been previously evaluated.

In this study, we rationally designed and characterized two CS hydrogels with a modified chimeric peptide (CP) sequence that is capable of promoting integrin recognition and neuritogenesis in NSCs. CS-A and CS-E glycosaminoglycans and CP constructs were functionalized with azide and DIBO biorthogonal handles [24, 54–56] to form SPAAC-crosslinked (CS-A)CP and (CS-E)CP click hydrogels. 3.5% CS hydrogels were used for functional testing as they resembled the biomechanical properties of brain tissue (600–1100 Pa) [38, 39, 57]. Furthermore, the amount of CS (∼33 nanomoles) in these hydrogels also closely mimicked the proportion of CS-A, which is the most predominant CS in the brain, i.e. about ∼1–63 nanomoles/gram brain tissue [46]. These characteristics make the CS-hydrogels attractive brain-mimetic tissue engineered constructs, that were further characterized *in vitro* and *in vivo,* in a novel rat CCI-SA model of sTBI. Cell–cell contact among NSCs is critical for survival, self-renewal and activation of MAPK signaling; which leads to enhanced NSC survival, proliferation, and neuronal differentiation [58, 59]. The greater migration and aggregation in hNSCs encapsulated in (CS-E)CP hydrogels is indicative of enhanced mechano-transduction and cell migration induced by FAK complex formation and activation [60, 61]. Based on these findings, we posit that (CS-E)CP hydrogels exhibit typical features of the adult stem cell niche, with enhanced aggregation and migration potential required for stem-cell maintenance [41, 62]. However, the ability of CS-E in supporting and maintaining neuronal differentiation has not been fully elucidated. Therefore, we tested the ability of the (CS-E)CP hydrogel in facilitating neuritogenesis and promoting spontaneous neural activity when compared to (CS-A)CP. We observed longer neurite extension and greater neuronal spiking activity of differentiated neurons on (CS-A)CP hydrogels when compared to those on (CS-E)CP hydrogels. These results are supported by previous findings demonstrating the axon outgrowth inhibiting attributes of CS-E. Interestingly, our results also indicate that, changes in neurite length do not influence neuronal activity, as revealed by the lack of any statistically significant differences in the mean firing rates of neurons on both hydrogels. Previous studies have demonstrated that CS-E binds selectively to the neurotrophic factor BDNF [47], suggesting that it can support neuronal activity. Other reports have also demonstrated that neurotrophic factor (FGF2 and BDNF) laden CS-matrices induced intralesional neurogenesis and functional recovery, chronically after TBI. Taken together, our results corroborate previous findings and further demonstrate that while integrin binding motifs in (CS-A)CP and (CS-E)CP can facilitate the migration, aggregation, and FAK complex formation of hNSCs; and neuritogenesis, and neuronal firing of differentiated neurons, differences in glycosaminoglycan sulfation can elicit distinct cellular responses that are potentially mediated by differences in growth factor signaling [58, 63], which remains to be elucidated.

We selected the (CS-A)CP hydrogels for subsequent intracavitary delivery after a CCI-SA sTBI due to the greater neuritogenic potential of (CS-A)CP hydrogels and the overall predominace of CS-A in the brain cortex. The CCI sTBI injury leads to hemorrhagic contusions and acts as a focal point for subsequent glial scarring, vascular disruption, and tissue loss. Therefore, the CCI-SA-sTBI model mimics resection of contused tissue to leave behind a tissue cavity, and the intracavitary delivery of 3D constructs represents a novel paradigm for assessing lesion volume deficits, cellular repair, biomaterial integration and cross-talk with native brain tissue. The (CS-A)CP hydrogel implanted animals demonstrated significantly accelerated recovery of forelimb motor function, 5-weeks after sTBI, which was likely mediated by the intralesional presence of neuroprotective and neuritogenic scaffolding [20, 21, 64, 65]. Immunohistochemical analyses of perilesional tissue confirmed that the hydrogel implant was able to prevent neuronal as well as axonal loss and demonstrated greater synaptic vesicle presence, indicating tissue recovery. We speculate that these outcomes were facilitated by previously demonstrated neuroprotective CS effects [14, 20, 21], as well as by CP driven neuritogenesis, which contributed to the significant improvement of reach-to-grasp function.

Axonal damage, neuronal loss, and excitotoxicity are essential components of the secondary injury cascade in sTBI [66, 67], and determining the status of intralesional neuronal circuitry is critical for functional assessments. However, we were unable to conduct deeper mechanistic investigations of these and other processes in the intracavitary implants due to a few key limitations. Although the hydrogel implants were found to be stably incorporated (Supplementary figure 4) in the gross brain images, post-mortem tissue preparation, staining, and washing procedures resulted in the significant loss of the intracavitary hydrogel implant. As a result, all analyses were restricted to perilesional quantifications. We believe that the size of the lesion cavity along with the relatively premature integration of the hydrogel implant with surrounding brain tissue at 5-weeks post-sTBI could have contributed to the loss of implanted material. Future investigations in longer duration studies might mitigate these issues by facilitating greater implant stability.

## 5. Conclusion

In summary, CS-hydrogels with varying sulfation patterns can be modified with biorthogonal ‘click’ handles to create functional arms carrying neuritogenic chimeric peptides. Our data provides evidence that these constructs facilitate neurite elongation and support neuronal activity, *in vitro*. CS-hydrogels were also able to accelerate functional recovery, *in vivo*, in a challenging sTBI model. This study opens up new avenues for using modified CS-hydrogels to enhance neuronal function, and accelerate tissue and functional repair and recovery after sTBI.

## Data Availability

All data needed to evaluate the conclusions in the paper are present in the paper and/or the supplementary files. Data can be obtained on demand by contacting the corresponding author: lohitash@uga.edu All data that support the findings of this study are included within the article (and any supplementary files).
